# Excess mortality in people with schizophrenia: 8-year population-based study in southern China

**DOI:** 10.1192/bjo.2025.10866

**Published:** 2025-10-23

**Authors:** Shaoling Zhong, Zihua Pan, Jinghua Su, Xiaoling Duan, Yanan Chen, Liang Zhou

**Affiliations:** Department of Community Mental Health, The Affiliated Brain Hospital, Guangzhou Medical University, China; Key Laboratory of Neurogenetics and Channelopathies of Guangdong Province and the Ministry of Education of China, Guangzhou Medical University, China

**Keywords:** Psychotic disorders/schizophrenia, mortality and morbidity, suicide, epidemiology, low- and middle-income countries

## Abstract

**Background:**

Schizophrenia is associated with premature mortality, but most evidence comes from high-income regions.

**Aims:**

This study aimed to estimate the excess mortality associated with schizophrenia in southern China.

**Method:**

We linked register data from a nationwide information system for psychosis to death registers. Individuals diagnosed with schizophrenia and residing in Guangzhou between 2014 and 2021 were included. Standardised mortality ratios (SMRs) were calculated to compare the mortality of people with schizophrenia with that of the general population. Life expectancy, potential years of life lost (PYLL) and years of life lost (YLL) were estimated for all-cause mortality and specific causes of death. Gender difference in these metrics was examined.

**Results:**

There were 3684 deaths (11.3%) during the study period. The leading causes of death were circulatory, neoplastic and respiratory diseases. The mortality rate among people with schizophrenia was twofold greater than in the general population, with a greater risk associated with unnatural causes than natural causes. The risk of mortality due to suicide was 15-fold higher than that of the general population. The life expectancy in schizophrenia was around 60 years, which is 21 years shorter than that for the general population. Schizophrenia was associated with substantial premature mortality burden, showing greater impact in men than women.

**Conclusions:**

Schizophrenia is associated with increased premature mortality, reduced life expectancy and substantial PYLL. The enduring disparity in mortality underscores an imminent call for targeted interventions aimed at suicide prevention and enhancement of the physical well-being of people with schizophrenia.

Schizophrenia is a chronic and severe mental disorder, affecting approximately 1% of the population worldwide.^1^ According to the Global Burden of Disease (GBD) study,^2^ the disorder remains a major cause of disability worldwide. People with schizophrenia have a two- to threefold higher risk of premature death, living 14.5 years less than the general population.^3^ The primary causes of excess mortality linked to schizophrenia are physical diseases, particularly respiratory diseases, cardiovascular diseases and cancers.^4^ Unnatural death, such as suicide,^5^ accounted for the majority of deaths.

Compared with relative measures of mortality, life expectancy – defined as the average number of years a person could expect to live at a given age – serves as an intuitive index of both mortality and health status, enabling comparisons across sub-populations. A growing body of studies^3,6^ using life expectancy as an alternative way to measure excess mortality have found a reduction in lifespan in people with schizophrenia. Life expectancy among people with schizophrenia is about 10–20 years less than in the general population, with men experiencing a greater reduction than women.^7^ Notably, recent studies have shown that, despite improvements in healthcare, this discrepancy in mortality has either remained static or increased.^3,8^ Therefore, health inequalities among individuals with schizophrenia represent a significant public health problem, and closing this mortality gap is recognised as a global health priority.

More evidence exists regarding mortality risk among people diagnosed with schizophrenia in high-income countries (HICs) and areas, particularly in Europe and North America, than in low- and middle-income countries (LIMCs) and areas.^3,9^ These studies may reflect wide mortality disparity, considering differences in healthcare resources, sociocultural contexts and population health indices across regions. Understanding the health consequences of schizophrenia in LMICs is crucial, because these countries account for over 85% of the world’s population and more than 80% of premature deaths from non-communicable diseases globally.^10^ Although studies focusing on LMICs are scarce, those available suggest that the mortality risk of schizophrenia in these areas is similar to, or even higher than, that in HICs. A systematic review involving 11 studies found that people with schizophrenia in Asia and Africa had the lowest life expectancy globally,^7^ although only 1 study from each region was included. Studies performed in LMICs have reported a standardised mortality ratio (SMR) ranging from 2 to 7.^11–13^ Furthermore, evidence remains limited regarding a comprehensive examination of specific causes of mortality and gender differences in schizophrenia in mainland China.

As evidenced by the findings discussed above, prior research has demonstrated significant heterogeneity in mortality risk patterns, life expectancy and years of life lost (YLL) across study populations,^3,7,12,13^ with particular variations observed between different geographic regions and healthcare settings. Thus, further research on mortality risk patterns within this specific population, particularly in LMICs, is warranted. In this 8-year population-based study, we aimed to estimate the premature mortality associated with schizophrenia in the community, using register data from a nationwide information system for psychosis. Specifically, we used three mortality metrics – SMR, life expectancy, potential years of life lost (PYLL) and YLL – to comprehensively measure the extent of excess mortality among people with schizophrenia compared with the general population. Additionally, we examined gender differences in excess mortality between men and women.

## Method

### Data source and study population

The study is reported following the Strengthening the Reporting of Observational Studies in Epidemiology guidelines (STROBE). We used patient data from the National Information System for Psychosis (NISP) of China. NISP is a nationwide electronic health database developed for community-based follow-up and treatment services for psychosis by the National Health Commission (NHC) of China. NISP is an integrated, electronic record database that captures clinical data across multiple mental health settings. The database includes a wide range of patient demographics and clinical information. Within the database, diagnoses are provided by psychiatrists according to either ICD-10 or ICD-11. Demographic and clinical data are collected and entered into the system by community-based mental health professionals. NISP includes only patients with schizophrenia who have consented to participate in community-based mental health management programmes. The system does not include patients who: (a) are not engaged with community mental health services or (b) remain undiagnosed. As of December 2019, more than 4 million individuals with schizophrenia were registered in NISP in China.^14^

In this study, we identified individuals with schizophrenia registered with NISP, covering all 11 districts in Guangzhou, China. Guangzhou is a major metropolitan city in Guangdong province, southern China, with a population of approximately 18.73 million. To obtain information on dates and causes of death, we linked NISP data with death registries maintained by the Centres for Disease Control and Prevention (CDC) in Guangzhou. The accuracy of mortality data was ensured by direct recording and verification of patients’ death status. Data on population statistics and registered deaths in Guangzhou, spanning each calendar year from 2014 to 2021, were obtained from CDC. Data linkage between NISP and death registries was performed using national identification numbers. Following successful linkage, all personal identifiers were removed and patients were assigned unique research identifiers for analysis, ensuring complete anonymisation.

The authors assert that all procedures contributing to this work comply with the ethical standards of the relevant national and institutional committees on human experimentation, and with the Helsinki Declaration of 1975 as revised in 2013. All procedures involving human subjects/patients were approved by the Institutional Ethical Board of the Affiliated Brain Hospital, Guangzhou Medical University (no. 2023-132). Due to the nature of the study, informed consent was waived.

### Outcomes

Causes of death were obtained from the national mortality registry, which is linked to the NISP database. Causes of death were categorised into natural and unnatural causes following the World Health Organization’s ICD-10 framework. Specifically, natural causes include infections and parasitic diseases (nos A00–99, B00–89, B91–4.9, B99); neoplasms (C00–97); blood and immune diseases (D50–89); endocrine diseases (E00–88); nervous system diseases (G00–4, G06, G08–93, G95–8); circulatory diseases (I05–9, I11, I20–7, I30–52, I60–9); respiratory diseases (J00–99); digestive diseases (K00–92); musculoskeletal system diseases (M00–99); genitourinary system diseases (N00–45, N47–96, N98); congenital malformations (Q00–99); and others. We categorised external causes (V01–Y89) as unnatural causes of death, including suicide deaths (X60–84).

### Statistical analysis

We calculated person-years for each individual from their NISP registration date until death or end of the study (31 December 2021), whichever occurred first. Crude mortality rates (CMRs) were calculated as the number of deaths divided by total person-years of observation, multiplied by 100 000. We calculated SMRs using indirect standardisation, adjusting for age, sex, and calendar year. The expected number of deaths was determined by applying stratum-specific mortality rates from the general population of Guangzhou to the corresponding person-years of the cohort. SMR was defined as the ratio of observed to expected deaths. We calculated 95% confidence intervals using an exact method based on Poisson distribution and computed two-sided *P*-values to test for statistical significance. Life expectancy was calculated using the abridged life table method, which estimates the total person-years lived within each 5-year age band of a cohort and then divides this sum by the total number of the cohort.

We also calculated PYLL to estimate premature mortality. A cut-off of 75 years for potential life expectancy was set, a commonly used threshold. For each death occurring before age 75 years of age, PYLL was calculated as the difference between 75 years and the age at death. For each cause of death category, both total and average PYLL were calculated. The 95% confidence intervals for average PYLL were estimated using bootstrap resampling with 1000 replications. Analyses were performed for the total population and stratified by gender. The Shapiro–Wilk test was used to test the normality of PYLL. Gender differences in life expectancy and PYLL across causes of death were examined using the independent samples *t*-test or Wilcoxon rank-sum, as appropriate. Bonferroni correction was applied to adjust *P*-values. In this analysis, only diseases with a sample size of five or more deaths were included for reporting of SMR and PYLL. As a result, the following diseases were included in the analysis: musculoskeletal system, blood and Immune diseases and congenital malformations. YLL were calculated based on the difference between age at death and expected age of death according to the GBD study,^10^ reflecting the years of life that patients would have lived if the corresponding life expectancy had been fulfilled. We calculated average YLLs with 95% confidence intervals for each gender and cause of death category using bootstrap methods.

To assess the potential impact of COVID-19 on mortality patterns, we conducted a sensitivity analysis comparing SMRs between the pre-COVID-19 period (2014–2019) and the COVID-19 period (2020–2021) for all causes of death. All statistical analyses were two-sided, with a significance level of 0.05, and were performed using Microsoft Excel 2021 for macOS and R version 4.3.2 for macOS (R Foundation for Statistical Computing, Vienna, Austria; see https://www.r-project.org/).

## Results

From 2014 to 2021, the number of people with schizophrenia registered with NISP in Guangzhou ranged from 18 225 to 27 173, with an annual average of 22 187. The study cohort comprised 32 600 individuals with schizophrenia, of whom 28 916 (88.7%) remained alive and 3684 (11.3%) died during the study period. Most people were men, middle-aged and resided in urban areas (Supplementary Table 1). The mean age at death for the total cohort was 63.1 years (s.d. 13.8). The main causes of death are presented in Table 1. A total of 88.25% of deaths were attributed to natural causes, including circulatory (35.18%), neoplastic (14.90%) and respiratory (14.39%).

**Table 1:** Causes of mortality in patients with schizophrenia by gender

Cause of death	Total		Women		Men	
	*N*	%	*N*	%	*N*	%
Circulatory	1296	35.18	624	38.90	672	32.31
Neoplasms	549	14.90	233	14.53	316	15.19
Respiratory	530	14.39	195	12.16	335	16.11
External causes	433	11.75	186	11.60	247	11.88
Others	302	8.20	129	8.04	173	8.32
Endocrine	231	6.27	122	7.61	109	5.24
Digestive diseases	173	4.70	56	3.49	117	5.63
Infections	56	1.52	12	0.75	44	2.12
Nervous system	48	1.30	17	1.06	31	1.49
Genitourinary system	46	1.25	17	1.06	29	1.39
Musculoskeletal system	9	0.24	8	0.50	1	0.05
Blood and Immune diseases	9	0.24	4	0.25	5	0.24
Congenital malformations	2	0.05	1	0.06	1	0.05
All causes	3684	100.00	1604	100.00	2080	100.00

The overall mortality rate in our sample was 2083.13/100 000 person-years, with 1918.55/100 000 person-years in women and 2230.69/100 000 person-years in men (Table 2). Mortality rates were highest for circulatory (CMR = 732.83/100 000 person-years), neoplastic (CMR = 310.43/100 000 person-years), respiratory (CMR = 299.69/100 000 person-years) and unnatural causes (CMR = 244.84/100 000 person-years). In this population, 169 suicide deaths were recorded (CMR = 95.56/100 000 person-years), with 99 in men (CMR = 106.17//100 000 person-years) and 70 in women (83.73/100 000 person-years).

**Table 2:** All-cause, natural-cause and unnatural-cause mortality in patients with schizophrenia by gender

Cause of death	Total			Women			Men		
	CMR	SMR	95% CI	CMR	SMR	95% CI	CMR	SMR	95% CI
Natural causes	1838.29	2.26	2.19–2.34	1696.08	2.34	2.22–2.47	1965.80	2.21	2.11–2.31
Endocrine	130.62	4.26	3.73–4.84	130.38	4.25	3.49–5.13	130.84	4.26	3.54–5.09
Nervous system	27.14	4.17	3.07–5.53	27.51	4.23	2.68–6.34	26.81	4.12	2.67–6.08
Digestive diseases	97.82	3.59	3.08–4.17	98.08	3.60	2.86–4.47	97.59	3.58	2.89–4.40
Infections	31.67	3.18	2.40–4.12	31.10	3.12	2.04–4.57	32.17	3.23	2.18–4.61
Respiratory	299.69	3.06	2.81–3.34	300.22	3.07	2.70–3.47	299.21	3.06	2.71–3.44
Circulatory	732.83	2.44	2.31–2.58	733.21	2.44	2.25–2.64	732.48	2.44	2.26–2.63
Genitourinary system	26.01	2.21	1.62–2.95	26.31	2.24	1.40–3.39	25.74	2.19	1.40–3.25
Neoplasms	310.43	1.14	1.04–1.24	310.99	1.14	1.00–1.29	309.94	1.13	1.01–1.27
Unnatural causes	244.84	9.41	8.55–10.34	222.48	10.04	8.65–11.59	264.89	8.99	7.90–10.18
Suicide	95.56	15.52	13.27–18.04	83.73	17.46	13.61–22.06	106.17	14.38	11.69–17.51
All causes	2083.13	2.49	2.41–2.57	1918.55	2.57	2.45–2.70	2230.69	2.43	2.32–2.53

CMR, crude mortality rate (per 100 000 person-years); SMR, standardised mortality ratio.

The SMR showed excess mortality in people with schizophrenia across all cause-of-death categories, with the exception of musculoskeletal system diseases and congenital malformations (Table 2). The SMR was higher for unnatural- than natural-cause death. The risk of death was 2.49 (95% CI: 2.41–2.57) times higher, with SMRs of 2.57 (95% CI: 2.45–2.70) for women and 2.43 (95% CI: 2.32–2.53) for men. The excess mortality associated with endocrine and nervous system diseases was elevated compared with the general population, with SMRs of 4.26 (95% CI: 3.73–4.84) and 4.17 (95% CI: 3.07–5.53), respectively. The risk of mortality from other natural causes was elevated one- to fourfold compared with the general population. Among unnatural causes, the risk of excess mortality related to suicide was 15-fold higher than the general population (SMR = 15.52, 95% CI: 13.27–18.04; women, SMR = 17.46, 95% CI: 13.61–22.06; men, SMR = 14.38, 95% CI: 11.69–17.51).

The life expectancy of the sample was 61.44 years (95% CI: 57.77–65.10). Compared with the general population, there was a reduction in life expectancy of approximately 21 years across all groups (women, −21.45 years; men, −21.42 years; total, −21.65 years). There was no difference in life expectancy between women and men (women, 63.82 years, 95% CI: 58.58–69.05; men, 59.46 years, 95% CI: 54.35–64.58; *t* = −1.03, *P* = 0.361).

The PYLL revealed excess mortality in people with schizophrenia (Table 3). In the cohort, the average PYLL (95% CI) for all-cause mortality was 13.31 (12.92–13.66) years. Unnatural causes contributed higher PYLL than natural causes (23.58, 95% CI: 22.28–24.87 *v*. 11.9, 95% CI: 11.59–12.32). The average PYLL due to suicide is 28.97 (27.24–30.66) years. Gender differences in PYLL across causes of death are presented in Table 3. Men demonstrated significantly higher PYLL in four major causes of death: circulatory diseases (11.9 *v*. 7.67 years, *P* < 0.001), endocrine and metabolic diseases (16.1 *v*. 9.81 years, *P* < 0.001), neoplasia (15.8 *v*. 12.9 years, *P* < 0.01) and respiratory diseases (9.69 *v*. 8.29 years, *P* < 0.01). For all other causes of death, gender differences were not statistically significant following adjustment for multiple comparisons.

**Table 3:** All-cause, natural-cause and unnatural-cause PYLL in patients with schizophrenia by gender

Cause of death	Total		Women		Men	
	Sum of PYLL	PYLL (95% CI)	Sum of PYLL	PYLL (95% CI)	Sum of PYLL	PYLL (95% CI)
Natural causes	38 784	11.9 (11.59–12.32)	14 024	9.89 (11.54–12.33)	24 760	13.52 (11.54–12.33)
Circulatory	12 799	9.88 (9.32–10.41)	4783	7.67 (6.91–8.41)	8016	11.93 (11.21–12.65)
Neoplasms	7989	14.55 (13.70–15.38)	3000	12.88 (11.37–14.22)	4989	15.79 (14.71–16.87)
Respiratory	4863	9.18 (8.33–10.01)	1616	8.29 (6.83–9.97)	3247	9.69 (8.74–10.64)
Endocrine	2948	12.76 (11.39–14.23)	1197	9.81 (8.07–11.61)	1751	16.06 (14.19–18.14)
Digestive diseases	2349	13.58 (11.99–15.23)	615	10.98 (8.23–13.82)	1734	14.82 (12.92–16.76)
Infections	1052	18.79 (15.66–22.03)	224	18.67 (13.25–24.91)	828	18.82 (15.18–22.54)
Nervous system	710	14.79 (11.36–18.44)	167	9.82 (4.83–15.41)	543	17.52 (13.42–21.74)
Genitourinary system	624	13.57 (9.78–17.19)	230	13.53 (7.00–20.17)	394	13.59 (9.45–18.14)
Unnatural causes	10 210	23.58 (22.28–24.87)	4091	21.99 (20.02–24.05)	6119	24.77 (23.19–26.49)
Suicide	5360	28.97 (27.24–30.66)	2180	28.68 (25.79–31.37)	3180	29.17 (27.06–31.49)
All causes	49 023	13.31 (12.92–13.66)	18 115	11.29 (10.72–11.85)	30 908	14.86 (14.40–15.36)

PYLL, potential years of life lost.

On average, the YLL per person for all patients with schizophrenia was 29.79 years (95% CI: 29.34–30.23). The YLL was higher among men (31.60 years, 95% CI: 31.11–32.10) compared with women (27.44 years, 95% CI: 26.82–28.06). Natural causes of death accounted for the majority of YLL (84.2%), with an average of 28.42 years (95% CI: 28.04–28.80) per person, while unnatural causes contributed 15.8% of total YLL with an average of 40.06 years (95% CI: 38.79–41.34) per person (Supplementary Table 2).

Sensitivity analysis revealed substantially higher mortality risk during the COVID-19 period compared with pre-COVID years (Supplementary Table 3). Overall SMR increased from 2.35 (95% CI: 2.26–2.44) in the pre-COVID period to 3.11 (95% CI: 2.93–3.30) during COVID-19, particularly for unnatural causes (SMR, 7.78 [95% CI: 6.95–8.68] to 21.38 [95% CI: 17.47–25.91]).

## Discussion

This study provides new data on mortality among people with schizophrenia in regions outside high-income countries, while also expanding the global database on mortality rates for individuals with schizophrenia living in the community. This population-based study offers quantitative data on the SMR, life expectancy, PYLL and YLL quantifying the extent of excess mortality among individuals with schizophrenia. Our results showed that excess mortality was observed in all cause of deaths, with a higher risk for unnatural- than for natural-cause mortality.

Notably, we found a high mortality rate due to unnatural causes, particularly suicide. The risk of excess mortality related to suicide was 15-fold higher than in the general population. The SMRs for suicide in our study were higher than the median SMRs of 12.9 for suicide reported in a systematic analysis,^15^ which examined 10 studies from earlier decades. The risk is higher than that reported in a Brazilian cohort study of psychiatric patients following their first psychiatric hospital admission,^16^ but comparable to findings in a 10-year follow-up study of a first-episode psychosis cohort in the UK.^17^ This disparity may be attributed to differences in study populations, such as clinical characteristics, or regional factors affecting mental health care access and treatment. Improvements in clinical management and the development of suicide prevention strategies should be considered to reduce suicide deaths in schizophrenia.^18^ However, the suicide rates reported in our study were not as high as those reported in other studies, which included a meta-analysis^19^ of suicide rates after discharge from psychiatric hospitalisation. The disparity may reflect the significant decline in suicide rates in China over recent decades, driven by factors such as urbanisation, improved living standards and reduced access to lethal means like pesticides.^20^ Cultural and social factors may also partially account for the difference.^21,22^

Our results, showing that people with schizophrenia have a higher risk of excess mortality compared to the general population, are particularly concerning because they corroborate a growing body of evidence that preventive measures have been far less effective in this patient population than in the general population. Individuals with schizophrenia had about twice the mortality of the general population. In particular, cause-specific SMRs ranged from 1 for neoplasms to 4 for endocrine disorders. The risk was consistent with previous evidence reporting a 2–3 times greater mortality risk than the general population in low- and high-income countries,^11,23^ as well as an earlier meta-analysis^24^ and a more recent meta-review.^9^ In keeping with previous studies in high-income countries,^25,26^ our results showed that endocrine had the highest SMR across all natural-cause mortality, with deaths related to circulatory, respiratory, and neoplasms among the main causes. Many studies attribute this pattern to antipsychotics side effects,^27^ unhealthy lifestyles,^28^ such as sedentary behavior^29^ and tobacco smoking,^30^ and reduced health-seeking behaviour.^31^ Additionally, a shared genetic disposition for schizophrenia and cardiovascular disease may also explain the association.^32^

In general, our study found that life expectancy for individuals with schizophrenia was 60 years. Our findings are consistent with prior research, which indicates that individuals with schizophrenia have a reduced life expectancy of more than 20 years. The overall excess mortality pattern observed in our study is in line with a systematic review that reported a weighted life expectancy of 64.7 years for individuals with schizophrenia.^7^ In contrast, the risk was lower than that reported in African studies, where a life expectancy of 46·3 was reported.^7^ Several explanations may explain this difference, including social, economic, and healthcare inequalities. Our YLL findings are consistent with prior international studies. For example, a Hong Kong population-based study found life-years lost of 23.01 years for men and 16.02 years for women with schizophrenia,^4^ while a Brazilian cohort reported an average YLL of 27.64 years in psychiatric patients.^16^ Higher estimates in our study may reflect methodological differences, regional healthcare factors, or the impact of COVID-19 during our study period. Our study did not detect sex difference in overall mortality, consistent Saha’s meta-analysis.^15^ However, in line with previous studies identifying greater PYLL for men than for women,^3^ we found that men are more prone to premature mortality from cardiovascular and metabolic diseases, although no sex difference in life expectancy was identified in our study.

### Implications

Our study highlights that the excess mortality rate among people with schizophrenia is a crucial concern. The increased risk of mortality and shortened life expectancy, particularly due to suicide and physical diseases, implies the need for suicide prevention and enhanced physical healthcare for this population. It is imperative that service providers raise awareness of healthcare gaps and increase knowledge about the strength of the excess mortality. There is accumulating evidence that the life expectancy gap is exacerbated by unequal healthcare.^33^ Physical symptoms may be overlooked and attributed, either entirely or partially, to psychiatric and psychological factors. For practitioners, health intervention that improve adherence to pharmacological and physical health management should be an important goal.^34^ Screening, monitoring, and treatment of physical diseases, particularly chronic diseases, in people with schizophrenia warrant immediate action.

### Limitations

Our study benefited from linkage with death registries, the inclusion of both genders, and mortality metrics-SMR, life expectancy, and PYLL. Yet, there are some limitations when interpreting our findings. First, our findings may not be generalisable to all individuals with schizophrenia, as the NISP only includes patients who have voluntarily consented to participate in community-based mental health management. It does not include patients who are not engaged with community mental health services or remain undiagnosed. Second, it is possible that the reported deaths may be an underestimate compared to the actual number. The NISP was linked to the CDC’s death registries in Guangzhou, which document deaths that within the city. There may be deaths of people with schizophrenia who moved out of the city and were lost to follow-up. Another potential limitation is the usual caveats associated with using data that is routinely gathered but not originally intended for research purposes,^35^ such as data from NISP and death registries. Our study focused on population-level mortality indicators rather than individual-level survival analysis. While we have previously demonstrated the application of time-to-event analysis techniques in this study,^36^ the current study did not employ such methods to account for differential follow-up durations. Future studies integrating both population-level indicators and individual-level survival analysis may provide more comprehensive insights into mortality patterns and risk factors in people with schizophrenia. Finally, our study period included the COVID-19 pandemic, which significantly affected mortality rates. We found that death rates increased during 2020–2021, especially for unnatural causes. This suggests our results should be interpreted with cautious.

In conclusion, our results suggested that people with schizophrenia exhibit heightened SMR, shortened life expectancy, and considerable premature mortality. The risk of mortality in schizophrenia was about 2-fold higher than the general population, with suicide showing the highest excess mortality. Our study highlights the need for suicide prevention and for improved physical healthcare for people with schizophrenia.

## Supporting information

Zhong et al. supplementary material Zhong et al. supplementary material

## Data Availability

The data-sets analysed in the current study are available from the corresponding author, L.Z., upon reasonable request.

## Supplementary material

The supplementary material is available online at https://doi.org/10.1192/bjo.2025.10866
