# Supplementary material for: Excess mortality in people with schizophrenia: 8-year population-based study in southern China
Source: BJPsych Open. 2025 Oct 23;11(6):e251. doi: 10.1192/bjo.2025.10866 (PMC12569625; doi:10.1192/bjo.2025.10866)
Supplement: Zhong et al. supplementary material [file S2056472425108661sup001.docx]

**Supplemental table 1. Sociodemographic characteristics in patients with schizophrenia.**

| **Characteristics** | | | **Alive**  **(n=28916)** | **Deceased**  **(n=3684)** | |
| --- | --- | --- | --- | --- | --- |
| **Age, mean (SD), years** | | | 51.3 (14.1) | 63.1 ± 13.8 | |
| **Age group, n (%)** | | |  |  | |
| <30 | 2098 (7.26%) | | | 52 (1.41%) | |
| 30-49 | 10194 (35.26%) | | | 531 (14.41%) | |
| 50-69 | 13869 (47.96%) | | | 1900 (51.57%) | |
| ≥70 | 2755 (9.53%) | | | 1201 (32.60%) | |
| **Gender, n (%)** | | |  |  | |
| Women | | | 14232 (49.22%) | 1604 (43.54%) | |
| Men | | | 14684 (50.78%) | 2080 (56.46%) | |
| **Residence, n (%)** | | |  |  | |
| Rural | | | 3882 (13.42%) | 848 (23.02%) | |
| Urban | | | 25034 (86.58%) | 2836 (76.98%) | |

**Supplemental table 2. All-cause, natural-cause, and unnatural-cause YLL in Patients with Schizophrenia by gender.**

| **Cause of death** | **Total**  **YLL (95%CI)** | **Women**  **YLL (95%CI)** | **Men**  **YLL (95%CI)** |
| --- | --- | --- | --- |
| **Natural causes** | 28.42 (28.04-28.80) | 26.00 (25.39-26.63) | 30.29 (29.79-30.82) |
| **Circulatory** | 26.28 (25.69-26.88) | 23.62 (22.80-24.45) | 28.74 (27.88-29.55) |
| **Neoplasms** | 31.35 (30.46-32.27) | 29.58 (28.11-30.88) | 32.66 (31.55-33.81) |
| **Respiratory diseases** | 25.29 (24.45-26.16) | 23.63 (21.97-25.25) | 26.26 (25.22-27.31) |
| **Endocrine** | 29.36 (27.89-30.76) | 26.36 (24.53-28.21) | 32.73 (30.68-34.97) |
| **Digestive diseases** | 30.25 (28.60-32.03) | 27.44 (24.54-30.41) | 31.59 (29.55-33.50) |
| **Infections** | 35.63 (32.40-38.82) | 35.47 (30.31-41.01) | 35.68 (31.94-39.75) |
| **Nervous system** | 31.34 (27.51-35.10) | 25.90 (20.02-32.59) | 34.33 (30.09-39.02) |
| **Genitourinary system** | 30.54 (26.85-34.49) | 30.47 (24.05-37.61) | 30.58 (26.41-35.29) |
| **Unnatural causes** | 40.06 (38.79-41.34) | 38.36 (36.04-40.45) | 41.33 (39.68-43.01) |
| **Suicide** | 45.46 (43.59-47.30) | 45.04 (42.12-47.61) | 45.75 (43.32-48.12) |
| **All causes** | 29.79 (29.34-30.23) | 27.44 (26.82-28.06) | 31.60 (31.11-32.10) |

Note: YLL, years of life lost.

**Supplemental table 3. Standardized mortality ratios for all-cause and cause-specific mortality in patients with schizophrenia before and during the covid-19 pandemic.**

| Cause of Death | Pre-COVID (2014-2019)  SMR (95% CI) | COVID period (2020-2021)  SMR (95% CI) |
| --- | --- | --- |
| Natural deaths | 2.10 (2.01-2.19) | 2.72 (2.55-2.91) |
| Endocrine | 4.08 (3.48-4.74) | 4.75 (3.64-6.09) |
| Nervous system | 4.52 (3.22-6.18) | 3.11 (1.42-5.91) |
| Digestive diseases | 3.25 (2.69-3.89) | 4.49 (3.36-5.87) |
| Infections | 3.04 (2.18-4.12) | 3.64 (2.04-6.00) |
| Respiratory | 2.70 (2.43-2.98) | 4.44 (3.75-5.23) |
| Circulatory | 2.18 (2.04-2.32) | 3.19 (2.89-3.52) |
| Genitourinary system | 1.92 (1.30-2.73) | 3.22 (1.80-5.30) |
| Neoplasms | 1.07 (0.97-1.18) | 1.34 (1.13-1.57) |
| Unnatural causes | 7.78 (6.95-8.68) | 21.38 (17.47-25.91) |
| All causes | 2.35 (2.26-2.44) | 3.11 (2.93-3.30) |

Note: SMR, standardized mortality ratio.
